# Relationship between Concentrations of Lutein and StARD3 among Pediatric and Geriatric Human Brain Tissue

**DOI:** 10.1371/journal.pone.0155488

**Published:** 2016-05-20

**Authors:** Jirayu Tanprasertsuk, Binxing Li, Paul S. Bernstein, Rohini Vishwanathan, Mary Ann Johnson, Leonard Poon, Elizabeth J. Johnson

**Affiliations:** 1 Jean Mayer USDA Human Nutrition, Research Center on Aging at Tufts University, Boston, MA, 02111, United States of America; 2 Moran Eye Center, University of Utah School of Medicine, Salt Lake City, UT, 84132, United States of America; 3 Institute of Gerontology, University of Georgia, Athens, GA, 30602, United States of America; University of Nebraska Medical Center, UNITED STATES

## Abstract

Lutein, a dietary carotenoid, selectively accumulates in human retina and brain. While many epidemiological studies show evidence of a relationship between lutein status and cognitive health, lutein’s selective uptake in human brain tissue and its potential function in early neural development and cognitive health have been poorly evaluated at a molecular level. The objective of this study was to evaluate the cross-sectional relationship between concentrations of brain lutein and StARD3 (identified as its binding protein in retinal tissue) among three age groups: infants (1–4 months, n = 10), older adults (55–86 years, n = 8), and centenarians (98–105 years, n = 10). Brain lutein concentrations were analyzed by high-performance liquid chromatography and StARD3 levels were analyzed by Western Blot analysis. The strong relationship in infant brains (*r* = 0.75, *P* < 0.001) suggests that lutein has a role in neural development. The relationship remained significant but weaker in older adults (*r* = 0.51, *P* < 0.05) and insignificant in centenarians (*r* = 0.08, *P* > 0.05), seven of whom had mild cognitive impairment (MCI) or dementia. These exploratory findings suggest an age-related decrease or abnormality of StARD3 activity in human brain. Given that StARD3 is also involved in cholesterol transportation, a process that is aberrant in neurodegenerative diseases, the potential protective function of lutein against these diseases remains to be explored.

## Introduction

Carotenoids are naturally occurring plant pigments which are commonly found in the diet. Of the 40 carotenoids found in common foods in the US, about 25 are measurable in human serum, tissues and milk, but only 2 carotenoids, lutein and zeaxanthin, cross the blood-retina barrier to form the macular pigment in the human retina [1][2]. The preferential accumulation of these two oxygenated carotenoids, also known as xanthophylls, in human retina can be explained by identification of three retinal carotenoid-binding proteins: 1) a lutein-binding protein, steroidogenic acute regulatory domain 3 (StARD3), 2) a zeaxanthin-binding protein, glutathione S-transferase P1, and 3) tubulin, which serves as a site for high capacity deposition of carotenoids in retina with less specificity than the other two binding proteins [3–5]. StARD3, previously known as MLN64, was first identified in malignant breast cancer [6]. King et al. found that StARD3 was also produced in glia and neurons in specific regions of human brain [7]. StARD3 is an integral membrane protein which resides in late endosome and lysosome organelles and is proposed to be involved in intracellular lipid (e.g. cholesterol) trafficking. This may be important in relation to cognitive function given that aberrations of cholesterol transport in brain are often linked to processes of aging and onset of neurodegenerative disorders including Alzheimer's disease [8].

Similar to the retina, lutein is also the predominant carotenoid in human brain tissue in early and late life [9–11]. While it accounts for 31% of total brain carotenoids in adults [10], lutein accounts for approximately 59% of total brain carotenoids in infants even with a brief exposure to a diet typically low in lutein [9]. We have reported that lutein and zeaxanthin in the macula are highly correlated with their concentrations in the brain [11][12]. Thus, macular pigment density likely reflects lutein and zeaxanthin brain concentrations. This may explain the epidemiological evidence of significant relationships between macular pigment density and cognitive health in three cross-sectional studies [13–15]. We have also reported that among the carotenoids, brain concentrations of lutein from autopsy specimens in older adults were most consistently related to pre-mortem measures of global cognition [10].

In order to better understand a possible role of lutein in cognitive function throughout the lifespan, a better biochemical understanding of the selective uptake of lutein in human brain tissue is needed. Therefore, the objective of this study is to evaluate the cross-sectional relationship between the concentration of lutein and StARD3 in brain tissue from infant, older adult and very old adult decedents.

## Materials and Methods

### Subjects

Voluntarily donated brain tissues from decedents from three age groups were studied: infants (1–4 months), older adults (55–86 years), and centenarians (98–105 years). Samples came from a subset of three studies previously published, and their characteristics have been previously described [9–11] and were obtained from the National Institute of Child Health & Human Development Brain & Tissue Bank for Developmental Disorders, University of Maryland (infants) (http://medscholl.umaryland.edu/btbank/), the National Disease Research Interchange, National Resource Center (older adults) and the Georgia Centenarian Study (centenarians). Tissues were obtained from various regions of the brain, which included hippocampus (Hipp), temporal (TC), frontal (FC), and occipital (OC) cortices. It is noteworthy that these regions of the brain are associated with memory (Hipp), auditory perception (TC), executive function (FC), and vision (OC). A total of seventeen tissues were obtained from infant decedents. Both FC and Hipp tissues were available from seven infant decedents, while three infant decedents provided only FC tissue. For older adults, each of eight decedents provided one OC tissue and one Hipp tissue, hence a total of sixteen tissues. Each of five centenarians provided both FC and TC tissues, while only FC tissue was acquired from four centenarians and only TC tissue from one centenarian. Tissues were identified using a unique numerical identifier which obscured the identity of the decedent. Tissues (~0.5 g) were stored at -70°C until analysis for carotenoids.

### Brain Carotenoid Extraction

The brain extraction procedure was adapted from Park et al [16]. The detailed extraction procedure has been described in the previous publication by Vishwanathan et al [12].

### Carotenoid Analysis by High Performance Liquid Chromatography

Brain extracts were analyzed using reverse-phase high performance liquid chromatography. Twenty microliters of the brain extract were injected onto a C30 carotenoid column (3 μm, 150 × 4.6 mm, YMC, Wilmington, NC). The method was described by Yeum et al. in detail [17]. The lower limit of detection is 0.2 pmol for carotenoids.

### StARD3 Western Blot Analysis

The brain tissues were homogenized in 10 mM Tris-HCl buffer (pH7.4) containing 0.2 mM PMSF and 10 μg/mL aprotinin to prepare total protein extracts. Proteins were separated on 4–15% gradient SDS–PAGE and transferred to 0.45 μm nitrocellulose membranes using a trans-blot SD semi-dry transfer cell (Bio-Rad, Hercules, CA) at 20 V for 1 h. Nonspecific binding was blocked by immersing the membrane in 5% (w/v) nonfat dried milk in 0.01% (v/v) Tween 20 in TBS for 1 h at room temperature on an orbital shaker. The membranes were rinsed briefly with two changes of TBS and incubated with primary antibody overnight. Primary antibody to StARD3 (rabbit polyclonal antibody, N-62 StAR) was a gift from Professor Walter L. Miller at the University of California—San Francisco, with a 1:5000 dilution ratio. After two changes of wash buffer, the membranes were incubated with goat anti-rabbit IgG (H+L)-HRP conjugate secondary antibody (1:1000) for 2 h at room temperature. To visualize bands, membranes were developed using ECL Plus Western blot detection reagents (Amersham Biosciences, Pittsburgh, PA). Images of western blots were captured by FluorChemQ gel image system, and the intensity of the bands was measured by AlphaView Q (Cell Bioscience Inc., CA).

### Statistical Analysis

Given the novelty of this work, no sample size calculations were feasible. Data are expressed as means ± SEM. Concentrations of *cis*- and *trans*-lutein were combined to yield total lutein concentrations. Band intensity of StARD3 measured by AlphaView Q (Cell Bioscience Inc., CA) was used to represent level of StARD3 from brain tissues. Differences in age between sexes among each age group were assessed using Wilcoxon rank sum test, while differences in age and body mass index (BMI, kg/m^2^) between older adult decedents and centenarians were assessed using Student's t-test. Differences in concentrations of lutein among the three groups of decedents were evaluated using one-way ANOVA, followed by pairwise comparisons with Bonferroni adjustments. Differences in concentrations of lutein and StARD3 level between two regions of the brain (only decedents with both brain regions) were evaluated using Wilcoxon signed rank test. Differences of StARD3 between brain tissues with and without detectable levels of *cis*-lutein, as well as differences of lutein between older adult decedents with normal cognitive function and Alzheimer’s disease were evaluated using Wilcoxon rank sum test. Pearson's correlation was performed to determine correlation between age and weight or height of infants, between age and BMI of adult decedents, and to determine if either brain lutein concentration and StARD3 level were related to interval between time of death and tissue storage. Pearson's correlation coefficients were also used to determine relationship between brain lutein and StARD3 level. Partial correlation analysis was used in multiple linear regressions. For the infant decedents, the multiple linear regression model included age, sex, and term status and for the older adults variables included age, sex, and presence of Alzheimer’s disease as potential confounders of the relationship between lutein and StaRD3. The significance level was set at *P* < 0.05. R version 3.1.1 was used for all statistical analysis.

## Results

### Subject Characteristics

**Table 1** describes the characteristics of the infant and adult decedents. Mean age of infant decedents was 95.6 days. Nine out of ten infant decedents ranged from 86 to 123 days, and one female infant decedent was 31 days at the time of death. There were two preterm infants both with gestational age of 36 weeks. Six out of ten infant decedents were male, and median age of male infants was marginally greater than the median age of female infants (P = 0.067). Eight infants were Caucasian, and two infants were African American. An infant who was the oldest at the time of death also had the highest height of 73.7 cm and the highest body weight of 9.5 kg among infant decedents. Height, but not weight, significantly correlated with age in infant decedents (*P* < 0.05). BMI was not calculated for infant decedents. Four infants died of SIDS while the remaining six infants died of various other conditions. (**S1 Table**). Because brain lutein concentration and StARD3 level were not significantly different between infants who died of SIDS and other causes, data from two groups were combined.

**Table 1:** Subject characteristics^a^.

	Infants^b^ (n = 10)	Older adults^b^ (n = 8)	Centenarians^b^ (n = 10)
**Age**^c^			
Mean (SEM)	95.6 (8.0)	76.4 (3.3)	100.2 (0.7)
Median	99.0	79.5	100.0
Range	31–123	55–86	98–105
**Preterm, n (%)**	2 (20%)	NA	NA
**Sex**			
Females, n (%)	4 (40%)	4 (50%)	10 (100%)
**Race**			
Caucasian, n (%)	8 (80%)	7 (87.5%)	8 (80%)
African American, n (%)	2 (20%)	0 (0%)	1 (10%)
Hispanic, n (%)	0 (0%)	1 (12.5%)	0 (0%)
**Height, cm or m**^d^			
Mean (SEM)	6.4 (0.4)	1.74 (0.02)	1.55 (0.02)
Median	6.1	1.73	1.57
Range	5.4–9.5	1.70–1.85	1.45–1.70
**Weight, kg**			
Mean (SEM)	6.4 (0.4)	81.0 (5.6)	55.6 (4.5)
Median	6.1	79.4	51.3
Range	5.4–9.5	52.2–97.5	33.6–77.1
**BMI, kg/m**^**2**^			
Mean (SEM)	NA	26.86 (2.18)	22.75 (1.34)
Median		27.47	22.84
Range		15.27–33.73	16.01–29.10
**Presence of disease, n (%)**			
Alzheimer’s disease	NA	3 (37.5%)	ND
MCI or dementia		ND	7 (70%)
Other		ND	9 (90%)
**Cause of death**			
SIDS, n (%)	4 (40%)	NA	NA
Others, n (%)	6 (60%)	8 (100%)	
**Time interval between death and tissue collection, hours**			
Mean (SEM)	16.8 (1.6)	13.9 (2.4)	ND
Median	17.5	11.0	
Range	9–23	7–24	
**Brain tissue sample, n**	17	16	15
Frontal cortex	10	0	9
Temporal cortex	0	0	6
Occipital cortex	0	8	0
Hippocampus	7	8	0

NA = not applicable

ND = no data available

^a^See S1–S3 Tables for more information

^b^One infant, one older adult, and one centenarian had no data for height and weight. One centenarian had no data for race.

^c^Age in days for infants, in years for older adults

^d^Height in centimeters for infants, in meters for older adults

There was a significant difference between mean age of older adults (range from 55 to 86 years) and mean age of centenarians (range from 98 to 105 years) (*P* < 0.0001). Among older adult decedents, the youngest subject also had the lowest BMI (55 yrs, 15.3 kg/m^2^), and the oldest subject also had the highest BMI (86 yrs, 33.7 kg/m^2^). There was a significant correlation between age and BMI (*P* < 0.01). However, there was no significant correlation between age and BMI in centenarians. BMI was not statistically different between older adults and centenarians. Half of the older adults were female, while all of centenarians were female. Mean age of older adult males was not significantly different from mean age of older adult females. Only one older adult subject was Hispanic, while the remaining seven subjects were Caucasian. Three subjects in the older adult group had Alzheimer’s disease, while only one centenarian had pre-mortem Global Deterioration Rating Scale higher than 3, which is classified as dementia. The remaining subjects in the centenarian group, three were classified as cognitive intact and six were classified as mildly cognitive impaired. There were no statistically significant differences in brain lutein concentration and StARD3 level between older adult decedents with normal cognitive function and Alzheimer’s disease; hence all data were analyzed together. Details of cause of death are available for older adults but not for centenarians (**S2 Table**). Details of presence of cancer, diabetes, and cardiovascular disease are available for centenarians (**S3 Table**). There were no significant relationships between post mortem interval (time of death to storage) and either brain lutein concentration or brain StARD3 level in both infants and older adults. Post mortem interval data was not available for centenarians.

### Brain Lutein Concentration

Mean concentrations of *trans*-lutein in brain tissues from each age group are shown in **Table 2**. *Trans*-lutein was detected in all brain tissues (range: 3.9 to 132.5 pmol/g in infants, 23.6 to 97.0 pmol/g in older adults, 12.9 to 258.1 pmol/g in centenarians). On the contrary, only five of 17 infant brain tissues, seven of 16 older adults tissues, and four or 15 centenarian tissues had detectable levels of *cis*-lutein. Both preterm infant decedents in this study had no detectable level of *cis*-lutein. Among brain tissues with detectable *cis*-lutein (ranging from 2.4 to 12.9 pmol/g), its concentration accounted for 3–9% of total lutein in infants, 4–10% in older adults, and 4–8% in centenarians (data not shown).

**Table 2:** Mean (±SEM) brain concentration of *trans*-lutein and StARD3 band intensity of infants, older adults, and centenarians.

Group	Brain region	*trans*-lutein (pmol/g)	P value*	StARD3 band intensity	P value*
Infants	FC (n = 10)	52.3 7± 14.95	0.938	7521.64 ± 1190.22	0.938
	Hipp (n = 7)	31.93 ± 6.27		6460.07 ± 1192.44	
	Overall (n = 17)	43.95 ± 9.53		7084.52 ± 839.85	
Older adults	OC (n = 8)	71.33 ± 7.36	0.039	2212.25 ± 352.89	
	Hipp (n = 8)	53.87 ± 6.27		1908.13 ± 339.80	
	Overall (n = 16)	62.61 ± 5.18		2060.19 ± 239.87	
Centenarians	FC (n = 9)	99.84 ± 23.57	0.625	8457.32 ± 805.98	0.125
	TC (n = 6)	94.48 ± 36.74		10887.65 ± 1695.23	
	Overall (n = 15)	97.70 ± 19.59		9429.45 ± 857.04	

* Significant difference between two regions of brain within each subject group. Wilcoxon signed-rank test was applied for only samples available from the same subject.

Among the three age groups overall means of *trans*-lutein concentration not sharing a common superscript are significantly different at *P* < 0.05 (univariate ANOVA with Bonferroni adjustment for multiple comparisons).

In the evaluation of each brain region separately, there were no significant differences between concentration of *trans*-lutein in FC and Hipp in infants, as well as between FC and TC in centenarians. However, the concentration of *trans*-lutein in OC was significantly greater than that in Hipp in older adults (Table 2, *P* < 0.05). Although there were no differences observed in concentrations of *trans*-lutein in Hipp between infants and older adults, and in FC between infants and centenarians, the mean lutein concentration of all brain tissues in centenarians was significantly greater compared to the mean concentration in infants (*P* < 0.05), and the mean value for older adults tissues was not significantly different from the these two groups. The same observation was also true for total lutein concentrations.

### Brain StARD3 Level

Level of StARD3 protein in brain tissue was reported as the intensity of the bands in western blot analysis (Table 2). There was no significant difference between StARD3 band intensity between FC and Hipp in infants, between OC and Hipp in older adults, and between FC and TC in centenarians. It is noteworthy that compared to infant brain tissues with non-detectable level of *cis*-lutein, infant tissues with detectable level of *cis*-lutein had significantly higher level of StARD3 (*P* < 0.05) (data not shown). There was no such significant difference in older adults and centenarians. Due to the nature of western blot analysis (separate runs were performed), the intensity of bands cannot be compared among subject groups.

### Relationship between Brain Lutein Concentration and StARD3 Level

**Fig 1:**
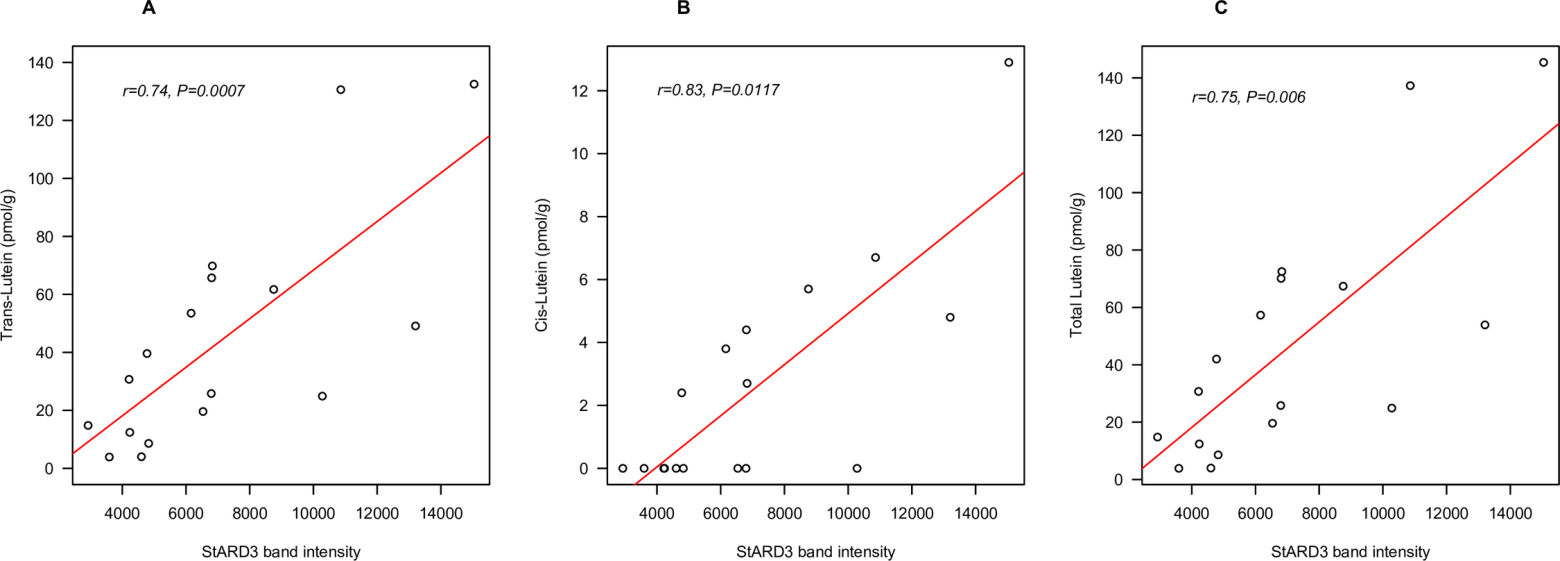
The relationship between *trans* (A), *cis* (B) and total (*trans*+*cis*) (C) lutein (pmol/g) and StARD3 (band intensity) in pediatric (1–4 months) decedents (n = 10). Tissues were from the frontal cortex (n = 10) and hippocampus (n = 7).

The correlations between brain lutein concentrations in the brain and StARD3 level were analyzed in each age group. *Trans*-lutein concentration in the brain of infant decedents was highly significantly related to StARD3 band intensity (*r* = 0.74, *P* < 0.001) (**Fig 1A**). When looked at separately for each region of the brain, the relationship was significant in FC (*r* = 0.82, *P* < 0.01) but not in the Hipp, perhaps due to the fewer samples from this region. *Cis*-lutein also significantly correlated with StARD3 in all infant brain tissues (**Fig 1B**, *r* = 0.83, *P* < 0.05) and in FC alone (*r* = 0.83, *P* < 0.01), and the significance of this correlation improved in all infant brain tissues when data points with undetectable level of *cis*-lutein were excluded (*r* = 0.79, *P* < 0.001). The correlation and significance of this relationship were also higher when both *cis* and *trans* isomers were combined (**Fig 1C**, *r* = 0.75, *P* < 0.001) than when evaluating *trans*-lutein alone. The relationship between total lutein and StARD3 remained statistically significant in all infant brain tissues after adjusting for covariate age and sex (*P* < 0.001), and also age, sex, and term status (preterm/full term) (*P* < 0.001).

**Fig 2:**
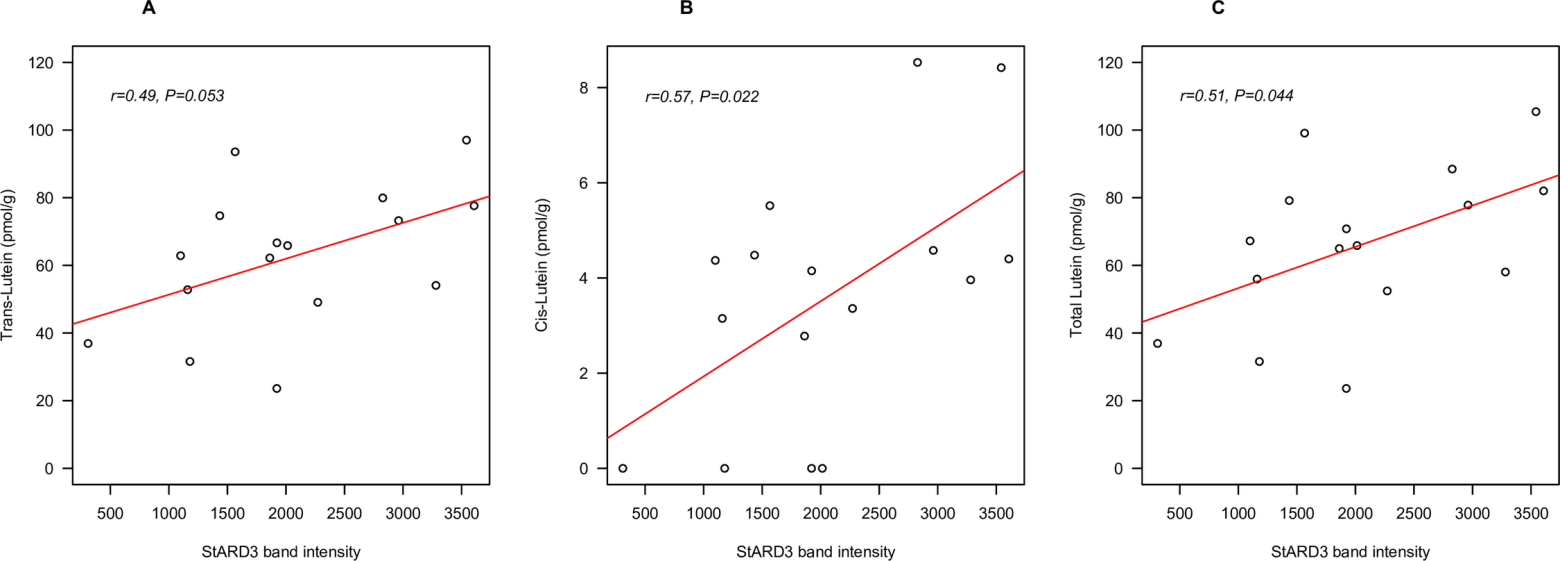
The relationship between *trans* (A), *cis* (B) and total (*trans*+*cis*) (C) lutein (pmol/g) and StARD3 (band intensity) in older adult (55–86 yrs) decedents (n = 16). Tissues were from the occipital cortex (n = 8) and hippocampus (n = 8).

**Fig 3:**
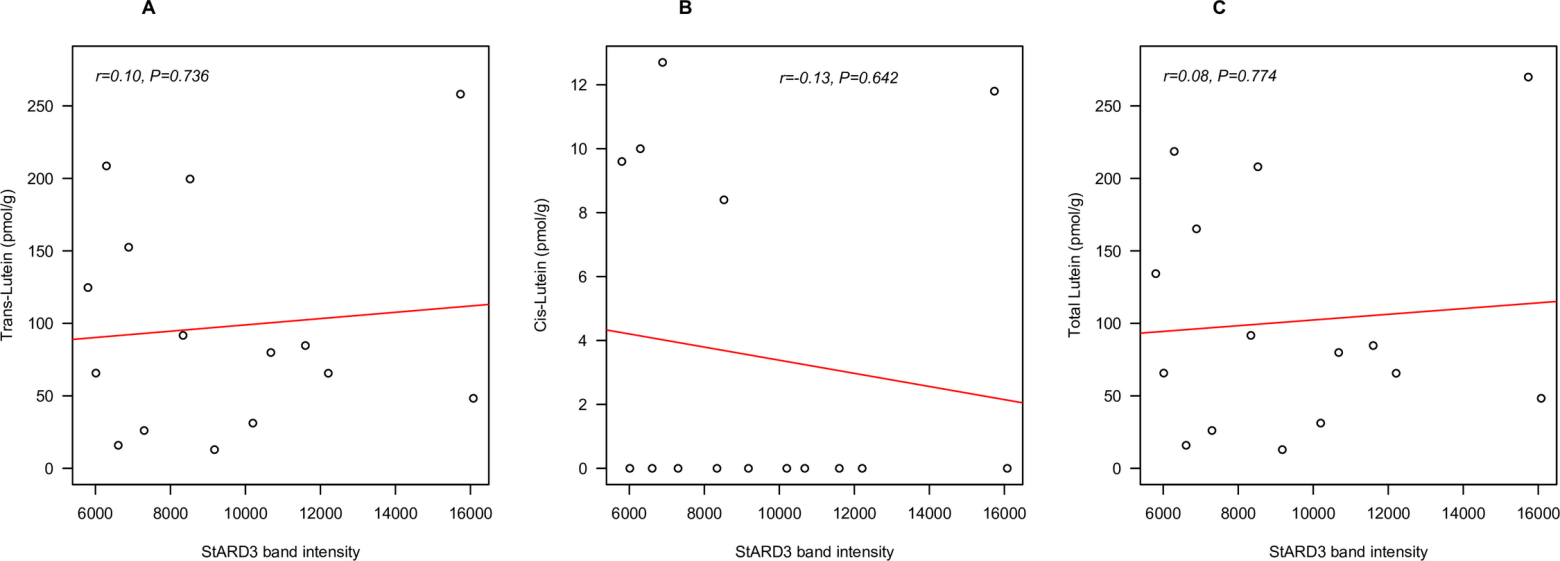
The relationship between *trans* (A), *cis* (B) and total (*trans*+*cis*) (C) lutein (pmol/g) and StARD3 (band intensity) in very old adult (98–105 yrs) decedents (n = 10). Tissues were from the frontal cortex (n = 9) and temporal cortex (n = 6).

In older adults, bivariate analysis showed a marginally significant relationship between brain *trans*-lutein concentration and StARD3 level (*r* = 0.49, *P* = 0.053) **(Fig 2A**). However, the relationships between StARD3 and *cis*-lutein alone (**Fig 2B,** r = 0.57, *P* < 0.05), and between StARD3 and total lutein (**Fig 2C**, r = 0.51, P < 0.05) were both significant. Data from OC or Hipp alone showed no significant relationship between lutein and StARD3. This was likely due to the low number of samples. Furthermore, the relationship between total lutein and StARD3 remained statistically significant after adjusting for presence of Alzheimer’s disease (*r* = 0.64, *P* < 0.05), and also after adjusting for covariates age, sex, and presence of Alzheimer’s disease (*r* = 0.60, *P* < 0.05). No significant relationships with StARD3 were found in centenarians for *trans*-lutein, *cis*-lutein or total lutein, (**Fig 3A–3C**). Excluding data points with undetectable level of *cis*-lutein did not improve any relationship.

## Discussion

StARD3 has been previously reported to be the specific binding protein for lutein in the retina and likely explains its selective accumulation in the macula [5][18]. As in the macula, lutein selectively accumulates in human brain tissue in early and later life [9–11]. This present study is the first to report cross-sectional relationships between brain lutein concentration and StARD3 level across the brain of three different age groups. Results from this study suggest that the preferential accumulation of lutein in brain tissue is due to this binding protein, as indicated by the significant relationship between lutein and StARD3 concentrations. However, the strength of the relationship declined with age. The relationship was highly statistically significant in tissues from infants, marginally significant in older adults, and not related in centenarians. In infant decedents, even after adjusting for age, sex, and term status (preterm/full term), the positive correlation between brain lutein and StARD3 remained highly significant. Based on the observation in our previous study that preterm infants had significantly lower concentration of lutein in their brain compared to term infants [9], term status was included in the model as a potential confounder. This strong relationship, along with the preferential accumulation of lutein among the carotenoids in infant brain, even with brief exposure lutein in the diet, suggests that lutein may be important in neural development during early life.

The age-related decrease in the relationship between lutein and StARD3 may imply that there is an age-related decrease/abnormality of StARD3 activity in the human brain. StARD3 is involved in cholesterol transportation [6], which is an essential process for various functions in neurons including myelination and *de novo* synthesis of steroids. Presence of Alzheimer's disease was included in the model for older adults based on the observation that after adjusting for AD status, the relationship between lutein and StARD3 became significant. Presence of AD is also a potential confounder of this relationship, though this may not reflect the reality, given that roles of lutein and StARD3 in the brain of AD patients are poorly understood. Indeed, three of eight older adults and seven of ten centenarian decedents had mild cognitive impairment or dementia. Therefore, the neurodegeneration that occurs with aging may explain the decrease in this relationship. In addition, this may also be an example of biological variability, which is more common in human subjects as age increases. Of interest would be to explore this relationship in cognitively intact centenarians to therefore aid in the differentiation between extreme aging and dementia. This would aid in the generalizability of our findings.

Improvement of the correlation with StARD3 when *cis*- and *trans*-lutein were combined compared to the *trans* isomer alone, together with a significant relationship between the *cis* isomer with StARD3 in infants and older adults, suggests potential binding of *cis*-lutein with StARD3. To date, there is no known report of *cis*-lutein binding activity of StARD3. In human retina, StARD3 has been recognized as a *trans*-lutein binding protein [1][5]. However, whether this suggests *cis*-lutein is important in neural development or cognitive health is still questionable given its very low level in human brain throughout life (4–10% of total carotenoids). Further, there appears to be a selective uptake of the *trans* isomer into the brain given that the *cis*:*trans* ratio is much lower in brain compared to matched serum [4]. Also, in many brain tissues there was an absence of *cis*-lutein.

Although the lutein/StARD3 relationship in brain tissue differed across the three age populations, lutein was the predominant carotenoid in all groups. The lower concentration of brain lutein in infants compared to older adults and centenarians is expected given that infants only have a brief exposure (<1 year) to foods rich in lutein compared to the old and oldest adults (>50 years). Nevertheless, it should be noted that the difference is only about two-fold. These findings further support a role for lutein in early neural development.

Although our study presents intriguing data to support a role for lutein in brain function, this present study holds the following limitations: 1) There is no direct evidence that StARD3 is a lutein-binding protein in human brain. However, it is likely to be, given that this protein was detected by the same antibody previously used, and was previously identified as lutein-binding protein in human retina [5][18]; 2) Only correlational data was presented. Therefore, it is difficult to determine whether the amount of lutein is determined by the level of StARD3, whether lutein increases level of StARD3 expression, or if StARD3 may even serve as a confounder in a relationship between lutein and other unknown factors; 3) The sample size in each group was small. However, this research was largely exploratory and the literature on lutein in brain tissue is quite new and few. Lutein’s relationships with the binding protein throughout the lifespan may direct future work evaluating a role for lutein in cognitive function. 4) Dietary lutein data was not available. Therefore, an examination of the possible impact of diet on these results was not possible. 5) This study assumes StARD3 as the only lutein-binding protein in human brain tissue, while in fact, it may not be. In human retina, although StARD3 binds specifically to lutein, another carotenoid-binding protein tubulin serves as a major deposit site of lutein, zeaxanthin, and *meso*-zeaxanthin [5]. This was not investigated in brain tissue.

This present study expands our knowledge of the preferential uptake of lutein in human brain tissue. Given that StARD3 is a specific deposition site of lutein in macula, its presence in the brain may explain the preferential accumulation of lutein in this tissue. Also, given that StARD3 is a membrane-associated protein, and lutein is also found in the membrane [19], lutein may be able to modulate functional properties and structural features of synaptic membrane, as it was shown to enhance gap junction communication between neurons [20]. As humans cannot synthesize lutein, a better understanding of transportation and uptake of lutein into brain may help us optimize nutritional interventions for early neural development and for people with high risk of neurodegenerative diseases, and to the extent of improving dietary recommendations in general population. Results from this descriptive and exploratory study, warrant further investigation of the molecular mechanisms of brain lutein and its role in neural development and delaying cognitive impairment.

## Supporting Information

S1 Table Characteristics of infants. C: Caucasian; AA: African American; SIDS: sudden infant death syndrome; ND = no data available.
(DOCX)

S2 Table Characteristics of older adults. C: Caucasian; ND = no data available; N: normal cognitive function; AD: Alzheimer’s Disease; COPD: chronic obstructive pulmonary disease; H: Hispanic; *Tissue thawed then refrozen before shipment to Tufts.
(DOCX)

S3 Table Characteristics of centenarians. CVD: cardiovascular disease; C: Caucasian; AA: African American; ND = no data available. *Dementia score: 0 = cognitively intact, 1 = mild cognitive impairment, 2 = dementia.
(DOCX)

## Abbreviations

BMI: body mass index
FC: frontal cortex
Hipp: hippocampus
MCI: mild cognitive impairment
OC: occipital cortex
StARD3: steroidogenic acute regulatory domain 3
TC: temporal cortex
