# Correction: Relationship between Concentrations of Lutein and StARD3 among Pediatric and Geriatric Human Brain Tissue

**DOI:** 10.1371/journal.pone.0159877

**Published:** 2016-07-18

**Authors:** Emanuela Jirayu Tanprasertsuk, Binxing Li, Paul S. Bernstein, Rohini Vishwanathan, Mary Ann Johnson, Leonard Poon, Elizabeth J. Johnson

There are two errors in Table 2. The P value in the “Older Adults” row is incorrectly omitted. In addition, superscripts denoting significant differences in the “*trans*-lutein (pmol.g)” column were incorrectly omitted. The authors have provided a corrected table here.

**Table 2:** Mean (±SEM) brain concentration of trans-lutein and StARD3 band intensity of infants, older adults, and centenarians.

Group	Brain region	*trans*-lutein (pmol/g)	P value*	StARD3 band intensity	P value*
Infants	FC (n = 10)	52.3 7± 14.95	0.938	7521.64 ± 1190.22	0.938
	Hipp (n = 7)	31.93 ± 6.27		6460.07 ± 1192.44	
	Overall (n = 17)	43.95 ± 9.53^a^		7084.52 ± 839.85	
Older adults	OC (n = 8)	71.33 ± 7.36	0.039	2212.25 ± 352.89	0.742
	Hipp (n = 8)	53.87 ± 6.27		1908.13 ± 339.80	
	Overall (n = 16)	62.61 ± 5.18^ab^		2060.19 ± 239.87	
Centenarians	FC (n = 9)	99.84 ± 23.57	0.625	8457.32 ± 805.98	0.125
	TC (n = 6)	94.48 ± 36.74		10887.65 ± 1695.23	
	Overall (n = 15)	97.70 ± 19.59^b^		9429.45 ± 857.04	

* Significant difference between two regions of brain within each subject group. Wilcoxon signed-rank test was applied for only samples available from the same subject.
