# Supplementary material for: Relationship between Concentrations of Lutein and StARD3 among Pediatric and Geriatric Human Brain Tissue
Source: PLoS One. 2016 May 20;11(5):e0155488. doi: 10.1371/journal.pone.0155488 (PMC4874591; doi:10.1371/journal.pone.0155488)
Supplement: S1 Table — C: Caucasian; AA: African American; SIDS: sudden infant death syndrome; ND = no data available. (DOCX) [file pone.0155488.s001.docx]

**S1 Table. Characteristics of infants**

| Age (d) | Preterm/ Full term | Gestational age (w) | Sex | Race | Height (cm) | Body weight (kg) | Cause of death | Time of death to tissue collection (hr) |
| --- | --- | --- | --- | --- | --- | --- | --- | --- |
| 123 | F | 40 | M | C | 73.7 | 9.5 | Drowning | 19 |
| 96 | F | 40 | M | C | 62.2 | 6.4 | Bronchopneumonia | 12 |
| 86 | F | 40 | F | C | 57.4 | 6.1 | SIDS | 23 |
| 98 | F | 40 | M | AA | 57.4 | 6.0 | Tetralogy of Fallot | 16 |
| 101 | F | 38 | F | C | 58.4 | 5.5 | SIDS | 22 |
| 118 | F | 40 | M | C | 64.8 | 7.3 | Asphyxia by suffocation | 19 |
| 31 | F | 41 | F | C | 54.6 | 5.4 | Cardiac arrhythmia | 9 |
| 108 | F | 40 | M | C | 59.7 | 6.1 | SIDS | 23 |
| 100 | P | 36 | M | AA | 59.7 | 5.4 | Dehydration | 14 |
| 95 | P | 36 | F | C | ND | ND | SIDS | 11 |

C: Caucasian; AA: African American; SIDS: sudden infant death syndrome; ND = no data available

**S2 Table. Characteristics of older adults**

| Age (y) | Sex | Race | Height (m) | Body weight (kg) | BMI (kg/m^2^) | Normal/ Alzheimer's disease | Cause of death | Time of death to tissue collection (hr) |
| --- | --- | --- | --- | --- | --- | --- | --- | --- |
| 78 | F | C | ND | ND | ND | N | Cancer | 7 |
| 55 | F | C | 1.85 | 52.2 | 15.25 | N | Lymphoma | 8.5 |
| 80 | M | C | 1.70 | 79.4 | 27.47 | AD | Heart disease | 20 |
| 80 | M | C | 1.78 | 92.5 | 29.19 | N | COPD | 24 |
| 80 | F | C | 1.70 | 79.4 | 27.47 | N | Pulmonary fibrosis | 21.5 |
| 73 | M | C | 1.73 | 88.9 | 29.70 | N | Large cell lymphoma | 12 |
| 79 | M | H | 1.75 | 77.1 | 25.18 | AD | Stroke | 8.4 |
| 86 | F | C | 1.70 | 97.5 | 33.74 | AD | Alzheimer's disease | 9.9 |

C: Caucasian; ND = no data available; N: normal cognitive function; AD: Alzheimer’s Disease; COPD: chronic obstructive pulmonary disease; H: Hispanic; *Tissue thawed then refrozen before shipment to Tufts

**S3 Table. Characteristics of centenarians**

| Age (y) | Sex | Race | Height (m) | Body weight (kg) | BMI (kg/m^2^) | Presence of diseases | | | |
| --- | --- | --- | --- | --- | --- | --- | --- | --- | --- |
|  |  |  |  |  |  | Cancer | Diabetes | CVD | Dementia* |
| 105 | F | C | 1.50 | 51.26 | 22.82 | Yes | No | Yes | 1 |
| 98 | F | C | 1.57 | 56.70 | 22.86 | Yes | No | Yes | 1 |
| 100 | F | ND | ND | ND | 21.70 | No | No | Yes | 1 |
| 100 | F | C | 1.57 | 68.49 | 27.62 | Yes | Yes | Yes | 1 |
| 100 | F | C | 1.70 | 77.11 | 26.63 | No | No | Yes | 1 |
| 99 | F | C | 1.55 | 69.85 | 29.10 | No | Yes | Yes | 0 |
| 102 | F | C | 1.45 | 33.57 | 16.01 | Yes | Yes | No | 1 |
| 101 | F | C | 1.57 | 48.53 | 19.57 | Yes | No | Yes | 0 |
| 99 | F | AA | 1.57 | 44.00 | 17.74 | No | Yes | Yes | 2 |
| 98 | F | C | 1.47 | 50.80 | 23.41 | No | No | No | 0 |

CVD: cardiovascular disease; C: Caucasian; AA: African American; ND = no data available

*Dementia score: 0 = cognitively intact, 1 = mild cognitive impairment, 2 = dementia
