# Supplementary material for: Relationship between Concentrations of Lutein and StARD3 among Pediatric and Geriatric Human Brain Tissue
Source: PLoS One. 2016 May 20;11(5):e0155488. doi: 10.1371/journal.pone.0155488 (PMC4874591; doi:10.1371/journal.pone.0155488)
Supplement: S2 Table — C: Caucasian; ND = no data available; N: normal cognitive function; AD: Alzheimer’s Disease; COPD: chronic obstructive pulmonary disease; H: Hispanic; *Tissue thawed then refrozen before shipment to Tufts. (DOCX) [file pone.0155488.s002.docx]

**S2 Table. Characteristics of older adults**

| Age (y) | Sex | Race | Height (m) | Body weight (kg) | BMI (kg/m^2^) | Normal/ Alzheimer's disease | Cause of death | Time of death to tissue collection (hr) |
| --- | --- | --- | --- | --- | --- | --- | --- | --- |
| 78 | F | C | ND | ND | ND | N | Cancer | 7 |
| 55 | F | C | 1.85 | 52.2 | 15.25 | N | Lymphoma | 8.5 |
| 80 | M | C | 1.70 | 79.4 | 27.47 | AD | Heart disease | 20 |
| 80 | M | C | 1.78 | 92.5 | 29.19 | N | COPD | 24 |
| 80 | F | C | 1.70 | 79.4 | 27.47 | N | Pulmonary fibrosis | 21.5 |
| 73 | M | C | 1.73 | 88.9 | 29.70 | N | Large cell lymphoma | 12 |
| 79 | M | H | 1.75 | 77.1 | 25.18 | AD | Stroke | 8.4 |
| 86 | F | C | 1.70 | 97.5 | 33.74 | AD | Alzheimer's disease | 9.9 |

C: Caucasian; ND = no data available; N: normal cognitive function; AD: Alzheimer’s Disease; COPD: chronic obstructive pulmonary disease; H: Hispanic; *Tissue thawed then refrozen before shipment to Tufts
