# Supplementary material for: Relationship between Concentrations of Lutein and StARD3 among Pediatric and Geriatric Human Brain Tissue
Source: PLoS One. 2016 May 20;11(5):e0155488. doi: 10.1371/journal.pone.0155488 (PMC4874591; doi:10.1371/journal.pone.0155488)
Supplement: S3 Table — CVD: cardiovascular disease; C: Caucasian; AA: African American; ND = no data available. *Dementia score: 0 = cognitively intact, 1 = mild cognitive impairment, 2 = dementia. (DOCX) [file pone.0155488.s003.docx]

**S3 Table. Characteristics of centenarians**

| Age (y) | Sex | Race | Height (m) | Body weight (kg) | BMI (kg/m^2^) | Presence of diseases | | | |
| --- | --- | --- | --- | --- | --- | --- | --- | --- | --- |
|  |  |  |  |  |  | Cancer | Diabetes | CVD | Dementia* |
| 105 | F | C | 1.50 | 51.26 | 22.82 | Yes | No | Yes | 1 |
| 98 | F | C | 1.57 | 56.70 | 22.86 | Yes | No | Yes | 1 |
| 100 | F | ND | ND | ND | 21.70 | No | No | Yes | 1 |
| 100 | F | C | 1.57 | 68.49 | 27.62 | Yes | Yes | Yes | 1 |
| 100 | F | C | 1.70 | 77.11 | 26.63 | No | No | Yes | 1 |
| 99 | F | C | 1.55 | 69.85 | 29.10 | No | Yes | Yes | 0 |
| 102 | F | C | 1.45 | 33.57 | 16.01 | Yes | Yes | No | 1 |
| 101 | F | C | 1.57 | 48.53 | 19.57 | Yes | No | Yes | 0 |
| 99 | F | AA | 1.57 | 44.00 | 17.74 | No | Yes | Yes | 2 |
| 98 | F | C | 1.47 | 50.80 | 23.41 | No | No | No | 0 |

CVD: cardiovascular disease; C: Caucasian; AA: African American; ND = no data available

*Dementia score: 0 = cognitively intact, 1 = mild cognitive impairment, 2 = dementia
